# Riclinoctaose Attenuates Renal Ischemia-Reperfusion Injury by the Regulation of Macrophage Polarization

**DOI:** 10.3389/fphar.2021.745425

**Published:** 2021-10-13

**Authors:** Yang Zhao, Zhao Ding, Wenhao Ge, Junhao Liu, Xi Xu, Rui Cheng, Jianfa Zhang

**Affiliations:** Center for Molecular Metabolism, Nanjing University of Science and Technology, Nanjing, China

**Keywords:** riclinoctaose, ischemia-reperfusion, immunomodulatory, macrophage polarization, DC-SIGN

## Abstract

Renal ischemia-reperfusion injury is a major trigger of acute kidney injury and leads to permanent renal impairment, and effective therapies remain unresolved. Riclinoctaose is an immunomodulatory octasaccharide composed of glucose and galactose monomers. Here we investigated whether riclinoctaose protects against renal ischemia-reperfusion injury. In mice, pretreatment with riclinoctaose significantly improved renal function, structure, and the inflammatory response after renal ischemia-reperfusion. Flow cytometry analysis revealed that riclinoctaose inhibited ischemia-reperfusion-induced M1 macrophage polarization and facilitated M2 macrophage recruitment into the kidneys. In isolated mouse bone marrow-derived macrophages, pretreatment with riclinoctaose promoted the macrophage polarization toward M2-like phenotype. The inhibitor of Nrf-2/HO-1 brusatol diminished the effects of riclinoctaose on macrophage polarization. In mice, intravenous injection with riclinoctaose-pretreated bone marrow-derived macrophages also protected against renal ischemia-reperfusion injury. Fluorescence-labeled riclinoctaose specifically bound to the membrane of macrophages. Interfering with mDC-SIGN blocked the riclinoctaose function on M2 polarization of macrophages, consequently impairing the renoprotective effect of riclinoctaose. Our results revealed that riclinoctaose is a potential therapeutic agent in preventing renal ischemia-reperfusion injury.

## Introduction

Renal ischemia-reperfusion injury (IRI) is a highly heterogeneous and common clinical syndrome, encompassing a spectrum of risk factors and affecting 13.3 million patients every year (Devarajan, 2006). IRI is a two-stage pathophysiological process characterized by restriction of blood supply to the kidney and restoration of blood and oxygen. The pathogenesis of renal IRI is complex and involves several mechanisms, including activation of neutrophils, oxidative stress, ATP and glycogen depletion, as well as cellular metabolic stress caused by mitochondrial dysfunction (Sharfuddin and Molitoris, 2011; Zuk and Bonventre, 2016; Gonsalez et al., 2019). In addition, several apoptotic pathways, including the intrinsic Bcl-2 family and regulatory p53 pathways, seem to be activated during renal IRI (Sharfuddin and Molitoris, 2011). These factors inevitably cause initial cell death and promote renal tubulointerstitial fibrosis in the chronic phase (Boor and Floege, 2015).

IRI is likely mediated by the immune cells in the kidney, and the predominant cell type is macrophage (Huen and Cantley, 2017; Yan et al., 2020). Macrophages play an essential role in driving the progression or repair of IRI, depending on their activation stage and phenotype (Huen and Cantley, 2017). Thus, ischemia/reperfusion (I/R)-related macrophage activations and inflammatory cytokine productions are critical to the graft dysfunction and even the occurrence of primary non-function. Increasing evidence supports the importance of M1/M2 polarization of macrophage in tissue injury and repair during IRI (Ricardo et al., 2008; Vinuesa et al., 2008). Current evidence indicates that heme oxygenases-1 (HO-1) induction can switch the phenotypic to an anti-inflammatory phenotype, M2 macrophages, and enhance phagocytic capacity, suggesting that HO-1 induction in macrophages is a potential therapeutic approach for IRI (Ferenbach et al., 2010; Bolisetty et al., 2017; Zhang et al., 2018).

There are several therapies in combat with IRI, including ischemic preconditioning, gene therapy, and pharmacotherapy (Gonsalez et al., 2019). Ischemic preconditioning consists of cycles of mild ischemic stress, which elicit tolerance against subsequent severe ischemia and is an essential tool for clinical management of IRI (Livingston et al., 2019). Gene therapy, such as RNA interference targeting transcription factors that drive inflammation and fibrosis, has also been reported to be therapeutically effective in experimental renal IRI (Haddad et al., 2021; Tang et al., 2021; Zhuang et al., 2021). The therapeutic efficacy of the RNA interference is dependent on its stability, which increases the difficulty and cost of clinical utility. Medication treatments for renal IRI mainly include the following. Designed pharmaceuticals such as doxycycline, levosimendan, and iloprost have been reported to reduce renal oxidative injury and facilitate repair (Kucuk et al., 2009; Ihtiyar et al., 2011). Leptin, ascorbic acid, and B12 act as natural antioxidants to protect against I/R-induced renal damage (Erkasap et al., 2004; Korkmaz and Kolankaya, 2009; Li et al., 2020). However, the available drugs have multiple limitations, and the development of effective and comprehensive therapeutic strategies is essential for treating IRI.

Succinoglycan is an exopolysaccharide produced mainly by a large number of soil microbes of *Agrobacterium*, *Rhizobium*, or *Pseudomonas* genera, etc. Succinoglycan oligosaccharide (riclinoctaose) is the depolymerized form of a specific succinoglycan, riclin, which has shown superior anti-inflammatory, anti-tumor activity, and immunogenicity (Yang et al., 2019; Yang et al., 2021). Riclinoctaose is a linear oligooctasaccharide, composed of one galactose and seven glucose residues, with a pyruvate group linked to the terminal glucose residue (Wang et al., 2021). Compared with other known functional oligosaccharides, riclinoctaose is an oligooctasaccharide rather than a mixture of oligomers with different degrees of polymerization. The amphipathic properties against water and low toxicity allow succinoglycan oligosaccharides to be used in improving the solubility and bioavailability of poorly soluble therapeutic agents (Choi et al., 2012). Our previous investigation has also shown that riclinoctaose modulates the composition and metabolism of gut microbiota and thus improves host health (Wang et al., 2021).

In the present study, we investigated the therapeutic effects of riclinoctaose on preventing renal IRI in mice. We demonstrated that riclinoctaose had a significant protective effect on renal IRI, which depended on the moue DC-SIGN (mDC-SIGN) of macrophages. Our results revealed that riclinoctaose is a potential therapeutic agent in preventing renal IRI.

## Material and Methods

### Chemicals and Materials

Riclinoctaose was an oligooctasaccharide depolymerized from a succinoglycan, riclin, and prepared and purified as described in our previous study (Wang et al., 2021). The structure was characterized by electrospray ionization mass spectrometry (ESI-MS), Fourier-transform infrared (FT-IR), mass spectrum (MS), ^1^H nuclear magnetic resonance (NMR), and ^13^C NMR spectroscopic methods. The molecular weight is 1,384. The purity of the compound was greater than 99.4%. The remaining components was traces of inorganic salts. Riclinoctaose was re-dissolved in 0.9% saline and filter sterilized (0.22 µm filters).

### Animals

Wild-type male C57BL/6J mice (6–8 weeks old, 18–22 g) were purchased from Model Animal Research Center of Nanjing University and housed in standard cages on a 12 h light/dark cycle and were allowed free access to food and water. All animal care and use procedures were in accordance with the guidelines of the Institutional Animal Care and Use Committee at Nanjing University of Science and Technology (ACUC-NUST-20190218).

### Cell Culture

Mouse macrophage cell line RAW 264.7 cells and mouse fibroblast cell line NIH-3T3 cells were maintained in DMEM (Gibco, Germany) supplemented with 10% fetal bovine serum (FBS) (Gibco, Germany) and 1% penicillin-streptomycin. Mouse hepatocyte cell line AML12 cells were cultured in DMEM/F12 medium (Gibco, Germany) supplemented with 10% FBS (Gibco, Germany), 1% Insulin-Transferrin-Selenium (Gibco, Germany), 40 ng/ml dexamethasone (Sigma Aldrich), and 1% penicillin-streptomycin. BMDMs were cultured in DMEM (Gibco, Germany), supplemented with 10% FBS, 20% L929-MCSF supernatant, and 1% penicillin-streptomycin.

### Renal I/R Model

Mice were anesthetized using isoflurane with oxygen (2%, 0.5 L/h). The core body temperature was maintained around 37°C by an electrically heated plate. The abdomen was shaved, and a midline incision was made to expose the renal hilum. Then, the bilateral renal pedicle was occluded by non-traumatic vascular clamps for 30 min. Following reperfusion, the peritoneum was closed with a 4/0 suture, and 0.5 mL sterile saline was administered intraperitoneally. The mice were placed into a ventilated incubator at 28°C for 2 h and then returned back to their home cages. 24 and 48 h after the bilateral renal reperfusion, mice were euthanized. Plasma and tissue samples were harvested for analysis of renal function.

### Histology and Immunohistochemistry

For histological analysis, tissues were fixed with 4% paraformaldehyde for 24–48 h, paraffin sectioned (5 μm), and stained with hematoxylin and eosin, with Periodic acid Schiff, or with Masson’s Trichrome. For immunohistochemistry, paraffin tissue sections were dewaxed and blocked with 3% goat serum, 1% BSA, 0.1% Triton X-100 in PBS for 1 h at room temperature. Sections were incubated with antibodies specific to BCL-2 (#ab692, Abcam), Bax (#14796, Cell Signaling), and F4/80 (#30325, Cell Signaling) at 4°C overnight, treated with TSA-biotin (1:50) and visualized with 2,2′-diaminobenzidine tetrahydrochloride. TUNEL staining was performed using the DeadEnd Fluorometric TUNEL System (Promega). Staining quantification was performed using ImageJ software.

### Biochemical Analyses

Serum creatinine and blood urea nitrogen were measured by commercial kits (C013-1-1 and C011-2-1, Nanjing Jiancheng, China). Relative LDH release was determined using a commercial kit LDH (BC0680, Solarbio, Beijing, China). The levels of renal malondialdehyde (MDA), superoxide dismutase (SOD), catalase (CAT), and glutathione peroxidase (GSH-Px) were measured using specific kits (A003-1, A001-1, A007-1, A061-1, Nanjing Jiancheng, China). Data were normalized to total protein concentration determined by the BCA protein assay kit (P0012S, Beyotime, China).

### Quantitative Real-Time PCR

Total RNA was extracted from snap-frozen renal tissue sections and isolated from BMDMs using Karrol reagent (Karroten, Nanjing, China), and subsequently converted to cDNA using reverse transcript kits (Karroten, Nanjing, China). mRNA level was analyzed by reverse transcriptase-PCR with SYBR green PCR master mix (Karroten, Nanjing, China). The expression of the target gene was normalized to mouse housekeeping gene *β-actin* and *Hprt*. Values are expressed as relative fold induction. Primer sequences are displayed in Table 1.

**TABLE 1:** Primer sequences for the quantitative real-time polymerase chain reactions.

Gene	Type	Sequence (5′-3′)
*β-actin*	Forward	GAT CAT TGC TCC TCC TGA GC
	Reverse	ACT CCT GCT TGC TGA TCC AC
*Hprt*	Forward	TCA GTC AAC GGG GGA CAT AAA
	Reverse	GGG GCT GTA CTG CTT AAC CAG
*Kim1*	Forward	CAA GTT AAA CCA GAG ATT CCC AC
	Reverse	CGT GAT GCT GAG AAG TCT CA
*Vimentin*	Forward	CTG GTT GAC ACC CAC TCA AA
	Reverse	CGT GAT GCT GAG AAG TCT CA
*Fibronectin*	Forward	CAA GTC GAT GCC ATC CCA G
	Reverse	CTG GCT GTA AAC CTG TAA TGG
*Tgfβ1*	Forward	TGC TTC AGC TCC ACA GAG AA
	Reverse	GTG GAT CCA CTT CCA ACC CA
*Tgfβ2*	Forward	ATT CCC AGC TTC TGG CTC AA
	Reverse	GTC ATG GTC CCA GCA CTC G
*Sod1*	Forward	CCA TTG AAG ATC GTG TGA TCT C
	Reverse	CTT GTT TCT CAT GGA CCA CC
*Sod2*	Forward	AAG GAA CAA GGT CGC TTA CA
	Reverse	AGC AGC GGA ATA AGG CCT GT
*Sod3*	Forward	CCT TCT TGT TCT ACG GCT TG
	Reverse	CTG GAC TCC CCT GGA TTT GA
*Gpx1*	Forward	GTC TGG GAC CTC GTG GAC
	Reverse	TTC TTG CCA TTC TCC TGG TG
*Bcl2*	Forward	AGG ATA ACG GAG GCT GGG AT
	Reverse	CTT CAG AGA CAG CCA GGA GA
*Bax*	Forward	CTG AGC TGA CCT TGG AGC
	Reverse	GAC TCC AGC CAC AAA GAT G
*P53*	Forward	TGC TCC GAT GGT GAT GGC CT
	Reverse	TGT GGC GAA AAG TCT GCC TG
*MCP-1*	Forward	GCT GGA GCA TCC ACG TGT T
	Reverse	ATC TTG CTG GTG AAT GTG TAG CA
*IL-1β*	Forward	GAG CTT CAG GCA GGC AGT ATC
	Reverse	GTA TAG ATT CTT TCC TTT GAG GC
*Icam*	Forward	ACT GAG GAG TTC GAC AGA AC
	Reverse	AGG ACC GGA GCT GAA AAG TT
*Tlr4*	Forward	GGT GAG AAA TGA GCT GGT AAA G
	Reverse	GCA ATG GCT ACA CCA GGA AT
*HO-1*	Forward	GGG TGA TAG AAG AGG CCA AGA
	Reverse	AGC TCC TGC AAC TCC TCA AA
*Arg-1*	Forward	CAT GGG CAA CCT GTG TCC TT
	Reverse	CGA TGT CTT TGG CAG ATA TGC A
*NQO-1*	Forward	AGC CAA TCA GCG TTC GGT AT
	Reverse	GTA GTT GAA TGA TGT CTT CT
*Il-10*	Forward	CTC TCT GCA AGA GAC TTC CA
	Reverse	CTC TCC GGA CTT GTG AAG TA

### Western Blot Analysis

Snap frozen kidneys were lysed in a radioimmunoprecipitation assay (RIPA) buffer. After total protein normalized (BCA protein assay kit, P0012S, Beyotime, China), Total protein of 20–40 µg/lane was separated by 8–10% SDS-PAGE gel electrophoresis and transferred onto 0.22 μm NC membranes. After blocking with 5% skimmed milk or 3% BSA diluted in Tris buffer saline plus 0.1% Tween 20 (TBST) for 1 h at room temperature, membranes were incubated overnight with β-actin (#20536-1AP, Proteintech), BCL-2 (#ab692, Abcam), and BAX (#14796, Cell Signaling). Then membranes were incubated 1 h with secondary antibody (Jackson Immuno Research, 1:10,000). Enhanced chemiluminescence (ECL) was performed using ECL kit (WB-12, Solarbio, Beijing, China), visualized and analyzed by Clinx ChemiCapture software (Clinx, Shanghai, China). Individual protein level was quantified by normalizing its intensity to the β-actin in the same sample and expressed relative to the levels of the respective control group, the mean of which was set as one.

### ^1^H NMR Sample Preparation and NMR Spectroscopy

Polar metabolites were extracted from the kidneys as described (Beckonert et al., 2007). Briefly, approximately 200 mg frozen kidney samples were homogenized in ice-cold solvent buffer (50% acetonitrile/50% water, 1 ml/200 mg tissue). After centrifugation at 12,000 g for 10 min at 4°C, the supernatant was collected and lyophilized. The polar tissue extracts were resuspended in 580 ml NMR buffer (100 mM sodium phosphate buffer, pH 7.4, in D2O, containing 0.5 mM TSP) and then centrifuged at 12,000g for 5 min. NMR spectra were recorded on a Bruker AVANCE III 500 MHz NMR spectrometer (Bruker GmbH, Karlsruhe, Germany) at 298 K.

### Spectra Processing and Data Analysis

All NMR spectra were manually adjusted for phase and baseline correction and peak alignment using Bruker Topspin 3.0 software (Bruker GmbH, Karlsruhe, Germany) by the same person. MestReNova (Version 8.0.1, Mestrelab Research SL) was used to convert ^1^H NMR spectra to ASCII files. The water signal and affected regions from 4.5 to 5.5 ppm were eliminated. The data were normalized to the total area of the remaining spectra. R software (http://cran.r-project.org/) was performed for multivariate statistical analysis. The NMR data were first analyzed using principal component analysis (PCA), partial-least-squares-discriminant analysis (PLS-DA), and principal coordinate analysis (PCoA) to obtain a general overview of the metabolic patterns. Orthogonal signal correction-partial least square discriminate analysis (OPLS-DA) was then performed to reveal differential metabolic changes in kidneys between two groups. Metabolites were identified by comparing NMR spectra with original database curated from published paper (Jarak et al., 2017; Mora-Ortiz et al., 2019a; Mora-Ortiz et al., 2019b) and software Chenomx NMR suite 7.7 (Chenomx Inc., Edmonton, AB, Canada). The fold-change values of metabolites and their associated *p*-values were calculated and visualized in colored tables.

### Isolation and Culture of BMDMs

BMDMs were isolated from C57BL/6J mice as described (Roach et al., 2001). In brief, mice were euthanized by cervical dislocation, and bone marrow was isolated from femurs and tibias using an aseptic technique. Erythrocytes were lysed in red blood lysing buffer on ice for 5 min. Progenitor cell suspensions were cultured for 7 days in macrophage complete medium (DMEM with 10% FBS, 100 U/mL penicillin, 100 μg/mL streptomycin, and 20% MCSF-containing L929 fibroblast culture supernatant). The adherent cells after 7 days in culture were macrophages.

### Macrophage Phagocytosis Assays

Strain-matched thymi were filtered through a 200-mesh screen to collect single cells, and the liberated thymocytes were stained with Cell Tracker Green CMFDA (40721ES60, Yeasen, Shanghai, China). Then, thymocyte apoptosis was induced by overnight culture in RPMI 1640 with the final 1 μmol/L of dexamethasone in (50–02–2, Sigma-Aldrich). BMDMs were seeded in a 24-well plate and incubated with Lyso-Tracker Red (L8010, Solarbio, Beijing, China) for 15 min. Macrophages were then overlaid with apoptotic thymocytes at a ratio of 10:1 for 1 h. After three times of washing with ice-cold PBS, colocalization between the red phagolysosome and green apoptotic thymocytes to represent phagocytosis using fluorescence microscopy.

### Intravenous Administration of BMDMs

The intravenous injections of BMDMs were implemented as described (Ferenbach et al., 2010). The BMDMs cells were digested using pancreatic enzymes without EDTA and collected by centrifugation at 2000 rpm and then washed three times with PBS. The cells were then counted and kept on ice until IV injection into animals *via* the tail vein 1 h before bilateral clamping of renal arteries. For the Nrf-2/HO-1 pathway blocking experiment, BMDMs were pretreated with brusatol (160 nM) for 1 h before treatment with riclinoctaose.

### Flow Cytometric Analysis

Whole kidney samples were collected 24 h after reperfusion and minced into small pieces (1–2 mm^3^) in ice-cold PBS and collected by centrifugation (500 g, 5 min). Collected tissue was incubated in collagenase 4 (1 mg/ml, Sigma-Aldrich) and DNAse 1 (0.1 mg/ml, Roche, Woerden, Netherlands) in RPMI 1640 medium for 1 h at 37°C with gentle agitation. After that, cells were triturated through a 70-µm cell strainer, centrifuged, and enriched. The isolated cells were counted after erythrocyte lysis (ACK lysis buffer, A1049201, ThermoFisher) and washed with RPMI-1640 cell culture medium for further analysis. Approximately 100,000 harvested cells from the kidney. Cells were incubated with FITC rat anti-mouse CD11b Clone M1/70 (eBioscience Cat#: 17–0112–82), PerCP rat anti-mouse Ly-6G Clone 1A8 (Biolegend Cat#: 127,653), APC rat anti-mouse F4/80 Clone BM8 (Biolegend Cat#: 123,115), PE rat anti-mouse CD206 (MMR) Clone C068C2 (Biolegend Cat#: 141,705) were used. The analysis was performed by a NovoCyte flow cytometer (ACEA Bioscience Inc. San Francisco, CA, U.S.A) and analyzed using NovoExpress 1.2.3 software (ACEA Bioscience). The gating strategy is showed in Supplementary Figure S1.

### Colocalization Experiment

Raw 267.4 cells were seeded to 35 mm glass-bottom microscope dishes (801,002, Nest) at a proper density and cultured overnight. The cells were cultured with media containing 200 μM riclinoctaose for 6 h, then incubated with fresh media for 1 h at 37°C, followed by incubating with the fluorescent lipophilic cell tracer of DiI (D8700, Solarbio) according to the manual methods. After three washes with PBS, confocal images were captured by a Nikon A1 confocal laser microscope (Nikon, Tokyo, Japan).

### Receptor Blockade

For receptor-blocking experiments, mannan (200 μg/ml), laminarin (200 μg/ml), Fcblock (10 μg/ml), or bartonella LPS (160 ng/ml) were preincubated with BMDMs for 1 h at 37°C prior to stimulation with riclinoctaose.

### Statistical Analysis

All data are expressed as means ± SD. The statistical analysis was performed using GraphPad Prism 8 (Graph Pad Prism Software Inc., San Diego, CA, United States). All the experiments were repeated at least 3 times, and each data set shown is the representative data. Replicate measurements were taken from distinct biological samples. Data were analyzed by the two-way ANOVA followed by Bonferroni post hoc analysis, and *p*-values <0.05 were considered significant.

## Results

### Pretreatment of Riclinoctaose Prevents Against Renal IRI

**FIGURE 1:**
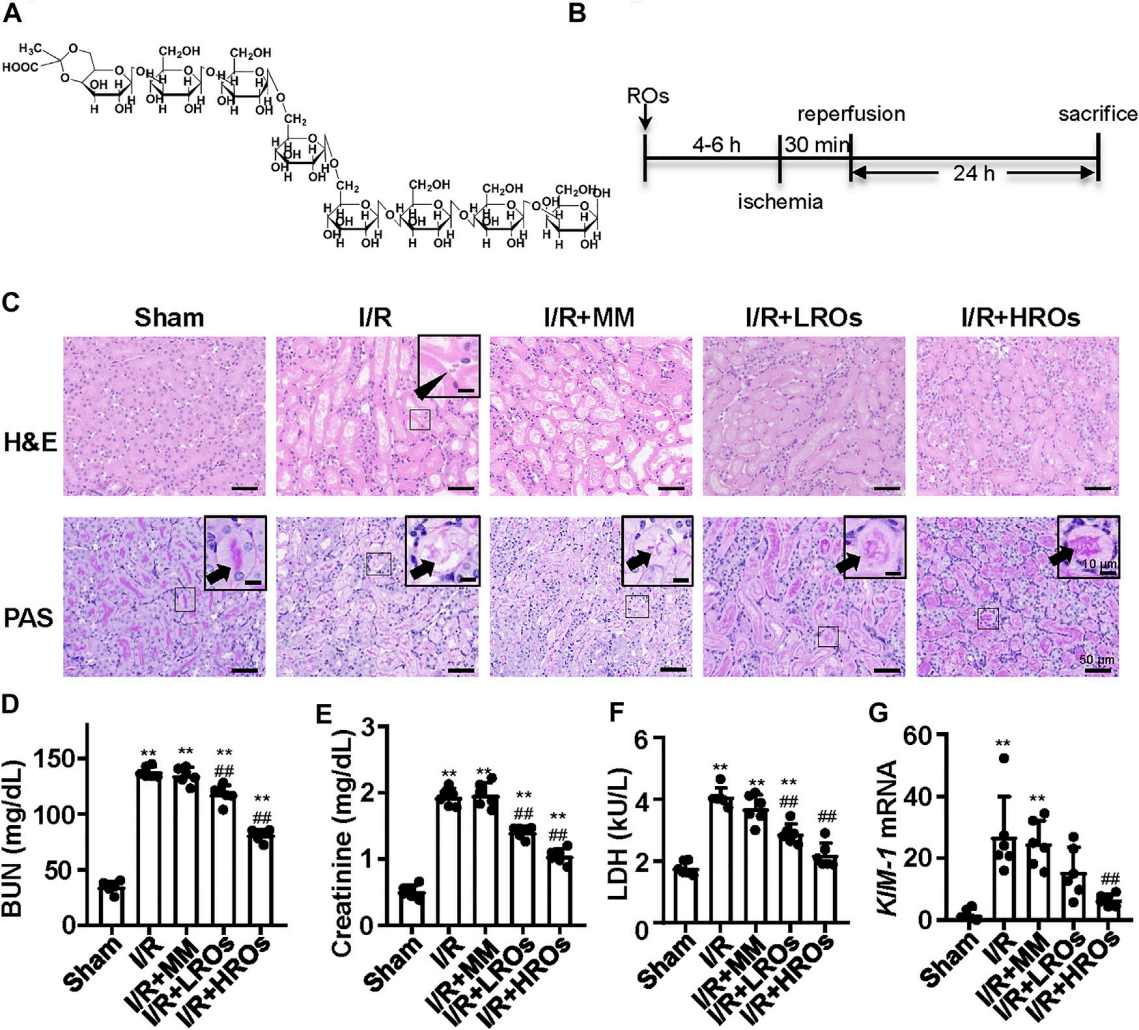
Functional and morphological protection from renal IRI with riclinoctaose at 24 h after reperfusion. **(A)** Chemical structure of riclinoctaose. **(B)** Schema for *in vivo* experiments. C57BL/6 mice underwent sham or I/R operation. I/R mice were treated with a peritoneal injection of vehicle, monosaccharide mix (MM: galactose 5 mg/kg and glucose 35 mg/kg), or riclinoctaose (LROs: 20 mg/kg; HROs: 40 mg/kg) 4–6 h before the 30 min-ischemia and sacrificed 24 h after reperfusion. **(C)** Upper panel: representative kidney section stained for hematoxylin and eosin (H and E) in each mice group. Arrowhead indicates cell debris. Lower panel: representative kidney section stained with Periodic acid–Schiff (PAS) in each mice group. Arrowheads indicate brush border. **(D-F)** Blood urea nitrogen (BUN) levels (D), serum levels of creatinine (Cre) (E), and lactate dehydrogenase (LDH) (F) in each mice group. **(G)** *Kim1* mRNA levels in kidneys of the four groups of mice. ROs: Riclinoctaose. Data are expressed as mean ± SD. n = 6, *p < 0.05, **p < 0.01 compared with the sham group; #*p* < 0.05, ##*p* < 0.01 compared with the I/R group. Data were analyzed by the two-way ANOVA followed by Bonferroni post hoc analysis.

Riclinoctaose is an octasaccharide with a stable pyruvate modification (Figure 1A). In order to examine whether riclinoctaose prevents renal IRI, the mice were pretreated with riclinoctaose 4–6 h before the 30 min-ischemia and sacrificed 24 h after reperfusion (Figure 1B). I/R resulted in the morphological alterations in the kidney, including tubular necrosis, intratubular cellular debris, and marked loss of the proximal tubular brush border. Pretreatment of riclinoctaose (LROs:20 mg/kg; HROs: 40 mg/kg) strikingly ameliorated IRI-induced histopathological characteristics with a dose-dependent manner (Figure 1C). Consistent with the histologic data, levels of blood urea nitrogen (BUN), creatinine, and lactate dehydrogenase were significantly increased in the kidneys of the I/R group compared with the sham group and riclinoctaose significantly alleviated the above changes (Figures 1D–F, Supplementary Figure 2A–C). The mRNA levels of *kidney injury molecule-1* (*KIM-1*) in the kidney were used as a metric to assess nephrotoxicity, which was increased ∼20x compared to the sham group at 24 h after reperfusion, and riclinoctaose groups LROs and HROs showed ∼34 and ∼70% lower than the I/R group, respectively (Figure 1G, Supplementary Figure 2D). These results indicated that pretreatment of riclinoctaose prevents against renal IRI in mice.

### Riclinoctaose Reduces I/R-Induced Kidney Fibrosis and Oxidative Stress

**FIGURE 2:**
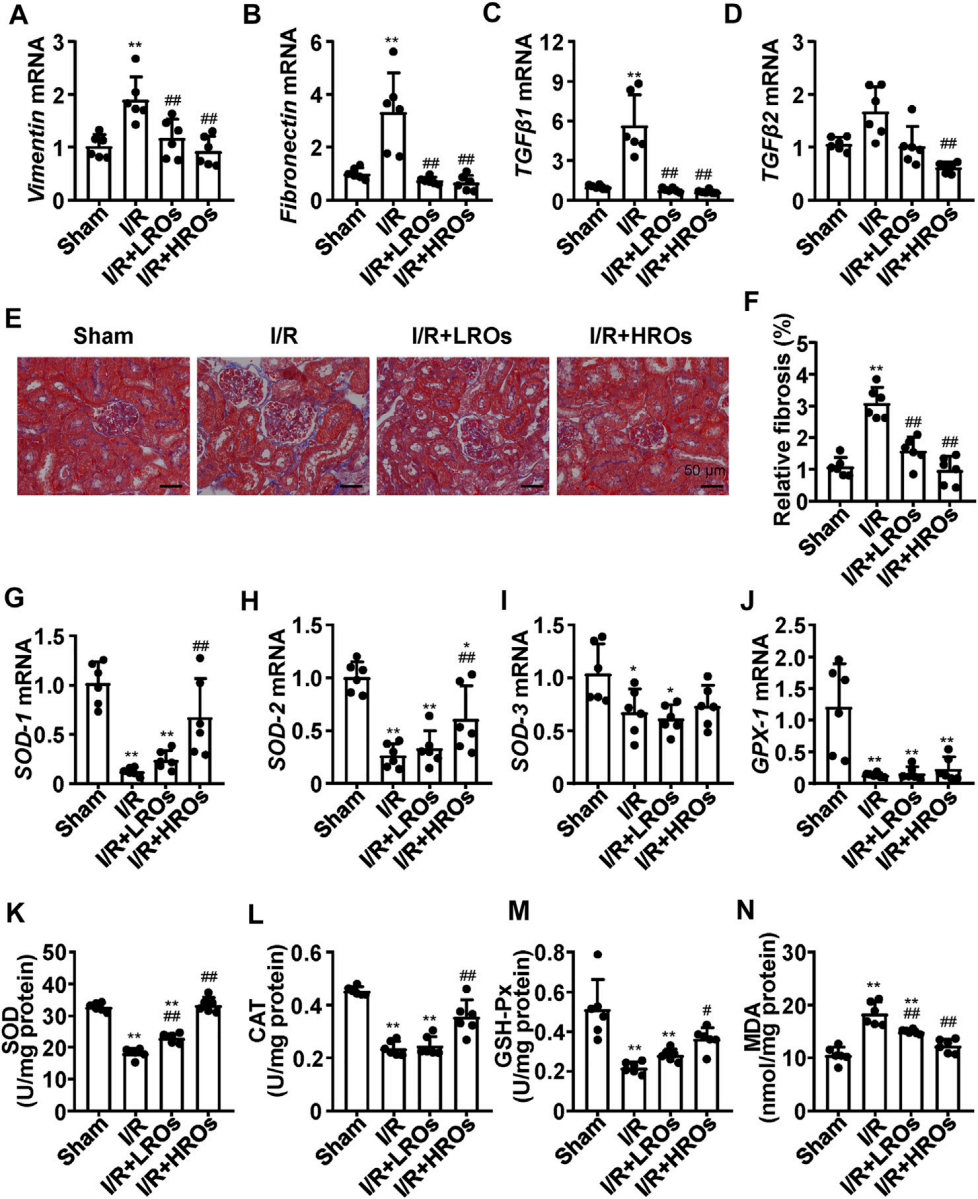
Suppression of I/R-induced fibrosis and oxidative stress with riclinoctaose. **(A–D)** mRNA levels of fibrotic genes, *Vimentin* (A), *Fibronectin* (B), *TGF-β1* (C), and *TGF-β2* (D) in kidneys of the sham group, I/R group, I/R + LROs group, and I/R + HROs group. **(E)** Representative Masson’s Trichrome stain of kidneys in each mice group. **(F)** Quantitative results of Masson staining. **(G–J)** mRNA levels of cellular antioxidant enzymes, *SOD-1* (G), *SOD-2* (H), *SOD-3* (I), and *GPX-1* (J) in kidneys of each mice group. **(K–M)** Antioxidant enzymes SOD (K), CAT (L), and GSH-Px (M) activities in the kidneys of each mice group. **(N)** Lipid peroxidation products MDA level in kidneys of each mice group. ROs: Riclinoctaose. Data are expressed as mean ± SD. n = 6, *p < 0.05, **p < 0.01 compared with the sham group; #*p* < 0.05, ##*p* < 0.01 compared with the I/R group. Data were analyzed by the two-way ANOVA followed by Bonferroni post hoc analysis.

Next, we examined whether riclinoctaose improves fibrosis in the kidney of I/R mice. While the mRNA levels of *Vimentin* and *Fibronectin* were markedly increased in the I/R group, riclinoctaose significantly decreased these gene expressions (Figures 2A,B). As shown in Figures 2C,D, the expression levels of *TGF-β1* and *TGF-β2* mRNA in the I/R group increased 6x and 1.5x compared with those in the sham group, pretreatment of riclinoctaose reduced these genes expression to the normal level. Consistent with the mRNA levels of various fibrogenic genes, histology showed higher levels of fibrosis within the tubulointerstitial areas in the untreated I/R mice but was markedly less in the kidneys of I/R mice pretreated with riclinoctaose (Figures 2E,F). Then, we found that the mRNA levels of cellular antioxidant enzymes, including *SOD-1*, *SOD-2*, *SOD-3*, and *GPX-1*, increased after riclinoctaose (Figures 2G–J). Moreover, while SOD, CAT, and GSH-Px enzyme activities were substantially reduced in the kidney of I/R mice, riclinoctaose significantly recovered the enzyme activities in a dose-dependent manner (Figures 2K–M). Riclinoctaose also attenuated the increases of renal MDA levels compared to those of I/R mice (Figure 2N). These results showed that riclinoctaose reduces I/R-induced kidney fibrosis and oxidative stress.

### Riclinoctaose Suppresses I/R-Induced Apoptosis

**FIGURE 3:**
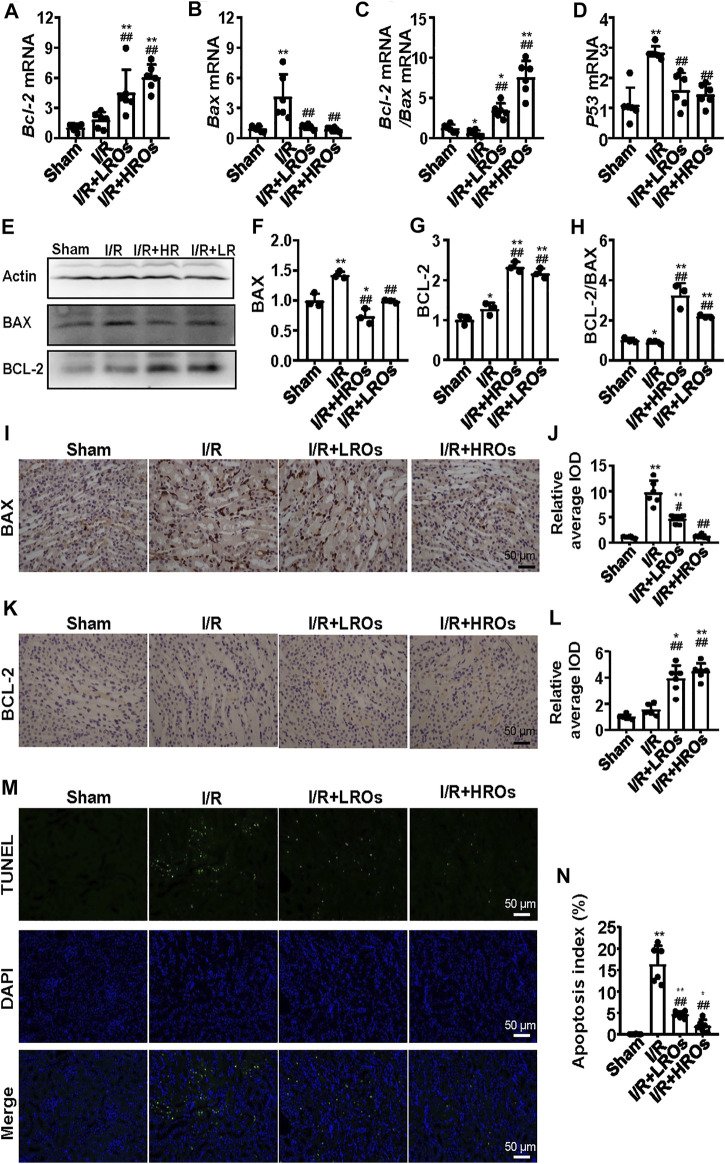
Suppression of I/R-induced apoptosis with riclinoctaose. **(A, B)** Relative mRNA expression of *Bax* (A) and *Bcl-2* (B) in the kidneys of the sham group, I/R group, I/R + LROs group, and I/R + HROs group. **(C)** The ratio of *Bcl-2* and *Bax* gene expression levels. **(D)** mRNA level of *p53* in the kidneys of the four groups. **(E–G)** Western blot and densitometric quantitation of BAX and BCL-2 in kidneys of four groups. **(H)** The ratio of BCL-2 and BAX protein levels. **(I, J)** Representative immunohistochemistry sections (I) and quantitative results (J) of BAX staining. **(K, L)** Representative immunohistochemistry sections (K) and quantitative results (L) of BCL-2 staining. **(M)** TUNEL-stain (green) indicates apoptosis-positive cells; DAPI stain (blue) indicates nucleated cells; the “merge” row shows cells stained with TUNEL and DAPI. **(N)** Quantitative results of TUNEL positive cells. ROs: Riclinoctaose. Data are expressed as mean ± SD. *n* = 6 for QRT-PCR, *n* = 3 for western blotting. *p < 0.05, **p < 0.01 compared with the sham group; #*p* < 0.05, ##*p* < 0.01 compared with the I/R group. Data were analyzed by the two-way ANOVA followed by Bonferroni post hoc analysis.

To further illustrate the protective effects of riclinoctaose on renal IRI**,** we analyzed I/R-induced apoptosis in riclinoctaose-treated mice. The mRNA level of *Bcl-2* (B cell lymphoma 2 apoptosis regulator) was slightly increased after I/R, and the increase was magnified to 6x by riclinoctaose-pretreatment compared to the sham group (Figure 3A). The mRNA levels of *Bax* (pro-apoptotic proteins bcl-2 associated X protein) were increased more than 4x in the kidneys of I/R mice, as compared to the sham group, and riclinoctaose-pretreatment reduced the extent of induction (Figure 3B). Correspondingly, treatment with riclinoctaose significantly upregulated the ratio of *Bcl-2*/*Bax* in I/R group kidneys from ∼0.6x to 7x compared to the sham group (Figure 3C). The mRNA levels of *p53* were increased 3x in the kidneys of untreated I/R mice compared to the sham mice, and riclinoctaose reduced the extent of induction (Figure 3D). Consistent with gene expression, the protein levels of BAX and BCL-2 increased, and the BCL-2/BAX ratio decreased in the kidney of I/R mice compared to that of sham mice (Figures 3E–H, left). Significantly, riclinoctaose caused a decrease of BAX, an increase of BCL-2, and an elevation of the ratio of BCL-2/BAX in a dose-dependent manner (Figures 3E–H, right). The immunohistochemical analysis confirmed the changes of these apoptosis-related proteins after riclinoctaose treatment (Figures 3I–L). Moreover, while I/R stress led to an increase in apoptosis rate, the number of TUNEL-positive cells was significantly decreased after riclinoctaose (Figures 3M,N). These results revealed that riclinoctaose suppresses I/R-induced apoptosis.

### Riclinoctaose Reverses the Shifts of Renal Metabolic Profiles of the Kidney in I/R Mice

**FIGURE 4:**
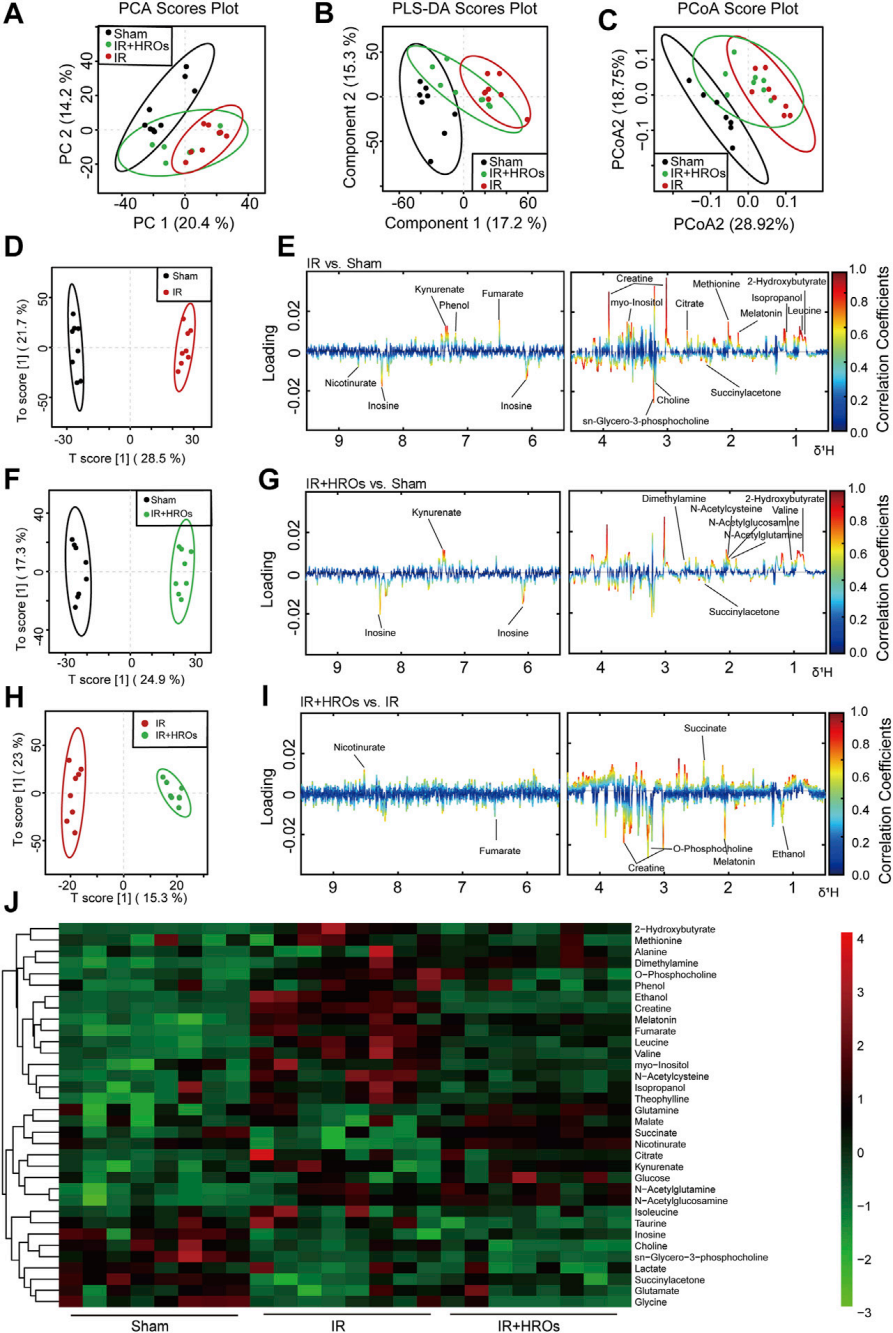
Effects of riclinoctaose on metabolomics in the kidney. **(A-C)** Score plots corresponding to the PCA analysis (A), PLS-DA analysis (B), and PCoA analysis (C) of polar metabolites from the sham group, I/R group, and high-dose riclinoctaose group. **(D, E)** Score plots and color-coded coefficient loadings plots corresponding OPLS-DA analysis of sham group and I/R group. **(F, G)** Score plots and color-coded coefficient loadings plots corresponding OPLS-DA analysis of sham group and high-dose riclinoctaose (I/R + HROs) group. **(H, I)** Score plots and color-coded coefficient loadings plots corresponding OPLS-DA analysis of the I/R + HROs group and I/R group. **(J)** Heat map of 34 metabolites from the sham group, I/R group, and I/R + HROs group. ROs: Riclinoctaose. *n* = 8. Symbols of solid black circles, solid red circles, and solid green circles represent the sham, I/R, and I/R + HROs groups, respectively.

We then examined the effect of riclinoctaose on the metabolic profile in the I/R kidney with ^1^H NMR-based metabolomics analysis. In the clustered score plots, the high dose riclinoctaose-pretreated group was deviated from the untreated I/R group and closed to the sham group, indicating that riclinoctaose effectively recovered metabolic abnormality induced by IRI (Figures 4A–C). In the OPLS-DA score plot and color-coded loading plots, the I/R group showed significant shifts of the metabolites (either increase or decrease) compared to sham groups (Figures 4D,E). However, the metabolite alterations were less pronounced between the riclinoctaose-treated I/R group and the sham group (Figures 4F,G). Riclinoctaose treatment reversed the metabolite alterations caused by IRI partially (Figures 4H,I). A heatmap based on the relative abundances of the metabolites was also generated to better display the separation in metabolites between the I/R group and the sham group, as well as the similarity in metabolites among the riclinoctaose-pretreated group and sham group (Figure 4J). The statistical analysis of major metabolites was performed and shown in Table 2. The significant elevations of the branched-chain amino acids (BCAAs), such as leucine, isoleucine and valine, in the kidneys of I/R mice suggested the enhanced protein degradations following IRI, which could be greatly ameliorated by riclinoctaose. Besides, the significantly increased levels of citrate and fumarate, and the obviously decreased levels of succinate in kidneys of the I/R group showed the turbulence of the TCA cycle, and such turbulence was reversed by riclinoctaose-pretreatment. The other significantly changed levels of metabolites in the I/R group, such as 2-hydroxybutyrate, isopropanol, ethanol, N-Acetylcysteine, succinylacetone, creatine, O-Phosphocholine, and nicotinurate were reversed after riclinoctaose pretreatment, suggesting good protection of riclinoctaose against IRI-induced metabolites disorder in mice. These results indicated that riclinoctaose reverses the shifts of renal metabolic profiles of the kidney in I/R mice.

**TABLE 2:** Identified metabolites in kidneys and their fold-change values and associated *p* values.

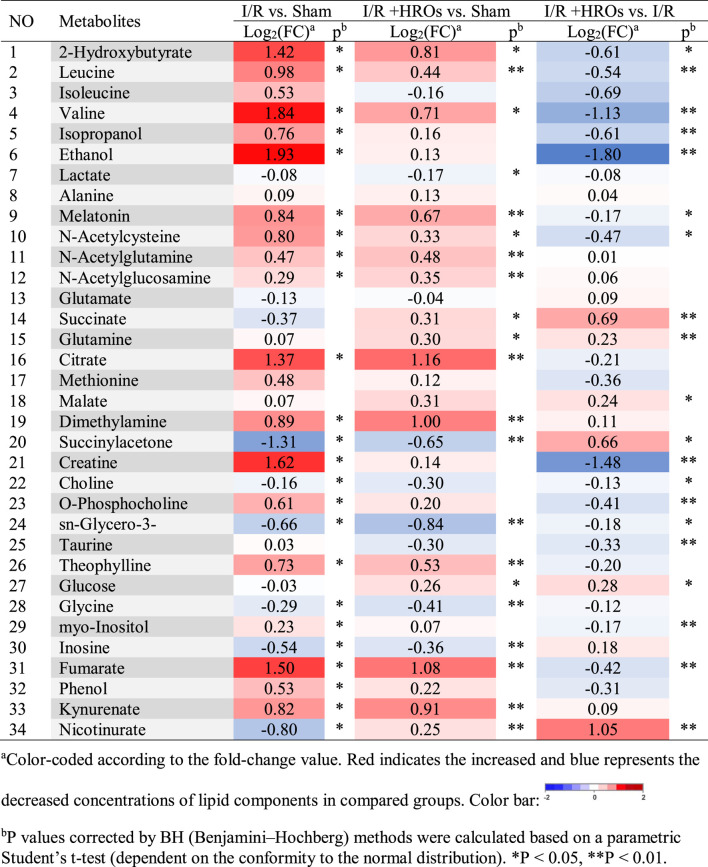

### Riclinoctaose Induces M2 Macrophage Polarization and Suppression of Myeloid-Derived Macrophage Infiltration

**FIGURE 5:**
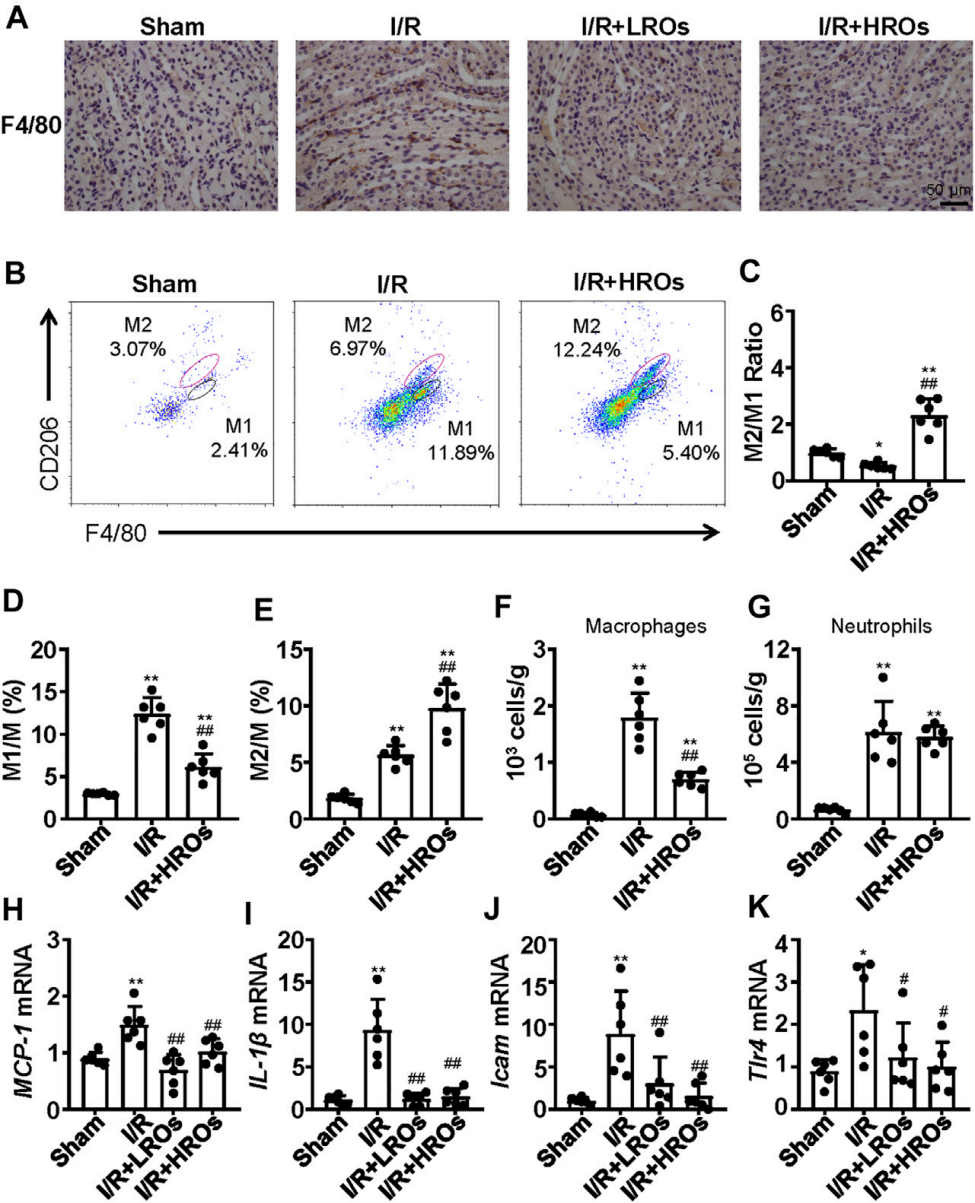
Effects of riclinoctaose on macrophage polarization and macrophage infiltration. **(A)** Typical immunohistochemical staining for anti-F4/80 of the kidneys in the sham group, I/R group, I/R + LROs group, and I/R + HROs group. **(B)** Representative plot chats of flow cytometry analyzing M1 and M2 macrophages after gating CD11b + Ly-6G-cells in the kidneys of the sham group, I/R group, and I/R + HROs group. M2 macrophages were defined as CD11b + Ly-6G-F4/80 + CD206-and M1 macrophages were defined as CD11b + Ly-6G-F4/80 + CD206+. **(C)** The ratio of M1/M2 macrophages in kidneys of the three groups. **(D, E)** The percentages of M1 macrophages and M2 macrophages in kidneys of the three groups. **(F, G)** The numbers of total macrophages and neutrophils were determined in the kidney of the three groups. **(H–K)** mRNA expression levels of *MCP-1* (H), *IL-1β* (I), *Icam* (J), and *Tlr4* (K) were examined by quantitative PCR in the kidneys of the sham group, I/R group, I/R + LROs group, and I/R + HROs group. ROs: Riclinoctaose. Data are expressed as mean ± SD. *n* = 6, *p < 0.05, **p < 0.01 compared with the sham group; #*p* < 0.05, ##*p* < 0.01 compared with the I/R group. Data were analyzed by the two-way ANOVA followed by Bonferroni post hoc analysis.

In order to determine whether the above protective phenotype of riclinoctaose on renal IRI is likely mediated by the macrophages, we investigated the effects of riclinoctaose on macrophage polarization and myeloid-derived macrophage infiltration in kidney tissues. Immunohistochemical staining analysis showed that an accumulation of F4/80+ myeloid-derived macrophages occurred in the kidney of mice 24 h after I/R, which was blocked by pretreatment with riclinoctaose (Figure 5A). Flow cytometry analysis indicated reciprocal changes of myeloid-derived macrophage (CD11b + F4/80+) polarization with decreased M1 macrophages (CD11b + Ly-6G-F4/80 + CD206-) and increased M2 macrophages (CD11b + Ly-6G-F4/80 + CD206+) after riclinoctaose (Figures 5B–E). We also observed an enhanced infiltration of myeloid-derived macrophages after I/R, and riclinoctaose significantly reversed these changes (Figure 5F). Riclinoctaose did not affect neutrophil infiltration after I/R (Figure 5G). The mRNA level of pro-inflammatory cytokines related to the M1 macrophage phenotype, including *MCP-1*, *IL-1β*, *ICAM*, and *Tlr4,* was significantly reduced after riclinoctaose (Figures 5H–K). Together, these data suggested that riclinoctaose promotes myeloid-derived macrophage polarization from the M1 to M2 phenotype.

### Intravenous Injection With Riclinoctaose-Treated Macrophages Improves Renal IRI

**FIGURE 6:**
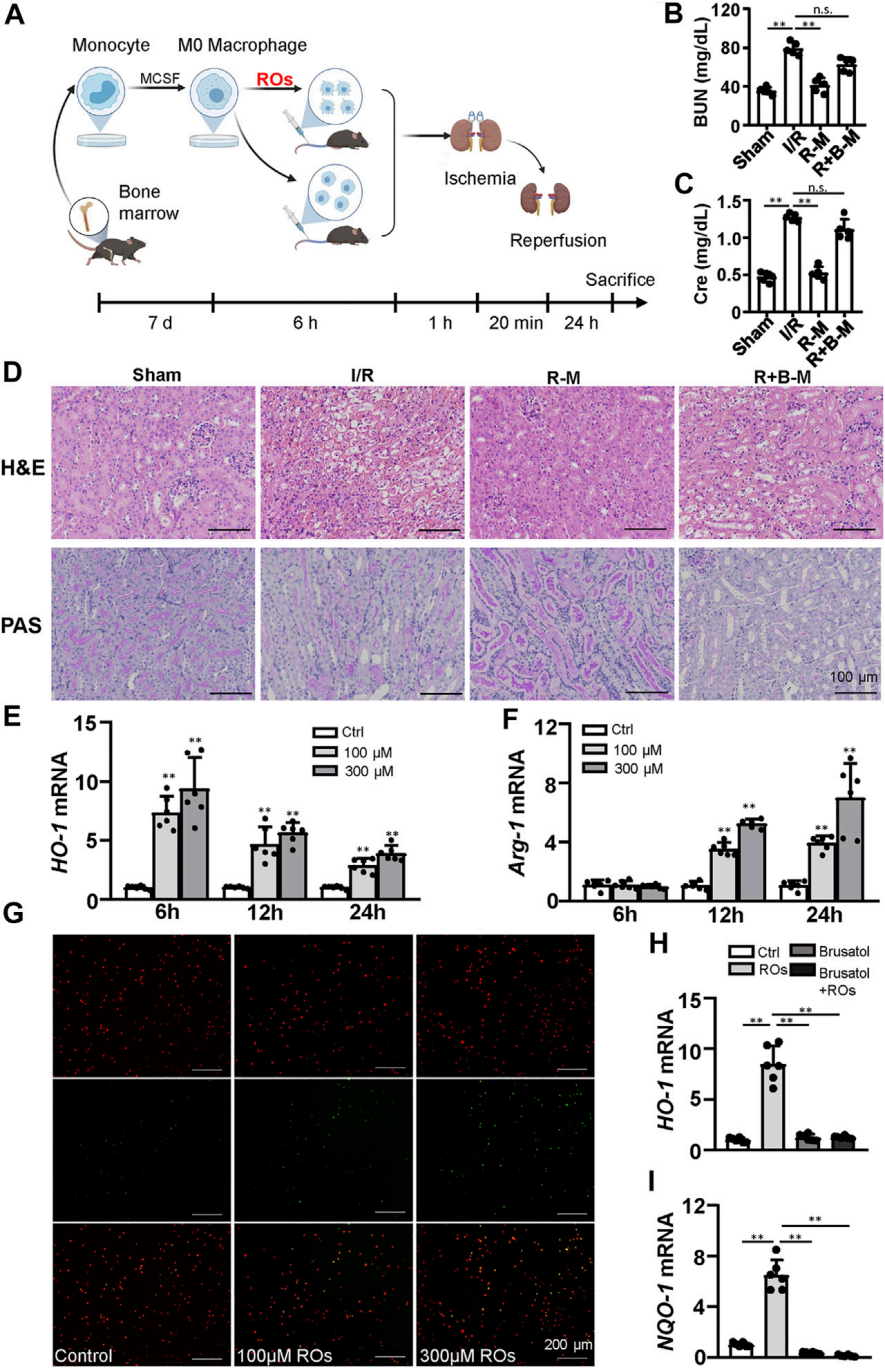
Macrophages mediate the renoprotective effects of riclinoctaose. **(A)** Schematic diagram of bone marrow-derived macrophages (BMDMs) injection strategy. BMDMs were isolated from femurs and tibias, polarized for 7 days, and stimulated with vehicle or riclinoctaose for 6 h. Then, treated BMDMs were injected intravenously 1 h before 20 min of bilateral ischemic, and mice were sacrificed at 24 h post-reperfusion. **(B, C)** Blood urea nitrogen (BUN) levels and serum levels of creatinine (Cre) in sham mice, mice injected with vehicle-treated BMDMs (I/R), mice injected with riclinoctaose-treated BMDMs (R–M), and mice injected with riclinoctaose and brusatol-cotreated BMDMs (R + B-M). **(D)** Upper panel: representative kidney section stained for hematoxylin and eosin in each mice group. Lower panel: representative kidney section stained with Periodic acid–Schiff in each mice group. **(E, F)** *HO-1* and *Arg-1* mRNA expression levels of BMDMs treated with riclinoctaose (100 and 300 μM) at different time points. **(G)** BMDMs (red) treated with vehicle or riclinoctaose for 6 h were incubated with apoptotic thymocytes (green) for 30 min, and the percent of phagocytized thymocytes was determined by fluorescent microscopy. **(H, I)** mRNA expression levels of *HO-1* and *IL-10* in BMDMs treated with riclinoctaose or brusatol alone or co-treated with riclinoctaose and brusatol. Ctrl: Control, ROs: Riclinoctaose. Data are expressed as mean ± SD. n = 6, *p < 0.05, **p < 0.01, n. s. nonsignificant. Data were analyzed by the two-way ANOVA followed by Bonferroni post hoc analysis.

To assess whether the effects of riclinoctaose on the myeloid-derived macrophage polarization contribute to the protection of renal IRI, we isolated the bone marrow-derived macrophages (BMDMs) and stimulated them with riclinoctaose for the indicated periods. BMDMs were aliquoted into three groups: vehicle, riclinoctaose, riclinoctaose plus brusatol. These treated BMDMs were injected intravenously 1 h before 20 min of bilateral ischemic (Figure 6A). Mice injected with riclinoctaose-treated BMDMs showed improved renal function (BUN and Creatinine) (Figures 6B,C). The H and E and PAS staining results confirmed the protective effects of riclinoctaose. (Figure 6D). Interestingly, a selective Nrf-2 inhibition brusatol blocked the effect of riclinoctaose on preventing renal IRI (Figures 6B–D). These results suggested that riclinoctaose improved renal IRI by altering macrophage polarization, likely through the activation of the Nrf-2 pathway. Nrf-2 is a transcription factor that increases the transcription of HO-1, which modulates macrophage polarization towards M2 macrophage. Then, we observed that riclinoctaose caused a significant increase of the mRNA level of *HO-1*, reaching a plateau at 6 h in BMDMs (Figure 6E). *Arginase 1* (*Arg-1*, a representative M2 marker) mRNA expression also increased significantly at 12 h after riclinoctaose and rose continuously until 24 h in BMDMs (Figure 6F). A dose-dependent enhancement of phagocytosis presented in riclinoctaose-treated BMDMs (Figure 6G). Moreover, riclinoctaose significantly elevated the expressions of *HO-1* and *NQO-1*, downstream factors of Nrf2 in BMDMs, and the effects of riclinoctaose on *HO-1* and *NQO-1* was entirely blocked by an Nrf-2 specific inhibitor brusatol (Figures 6H,I). Taken together, these results revealed that the protective effect of riclinoctaose on renal IRI is through the Nrf-2/HO-1 modulated macrophage polarization.

### Interfering With mDC-SIGN Blocked the Riclinoctaose Function

**FIGURE 7:**
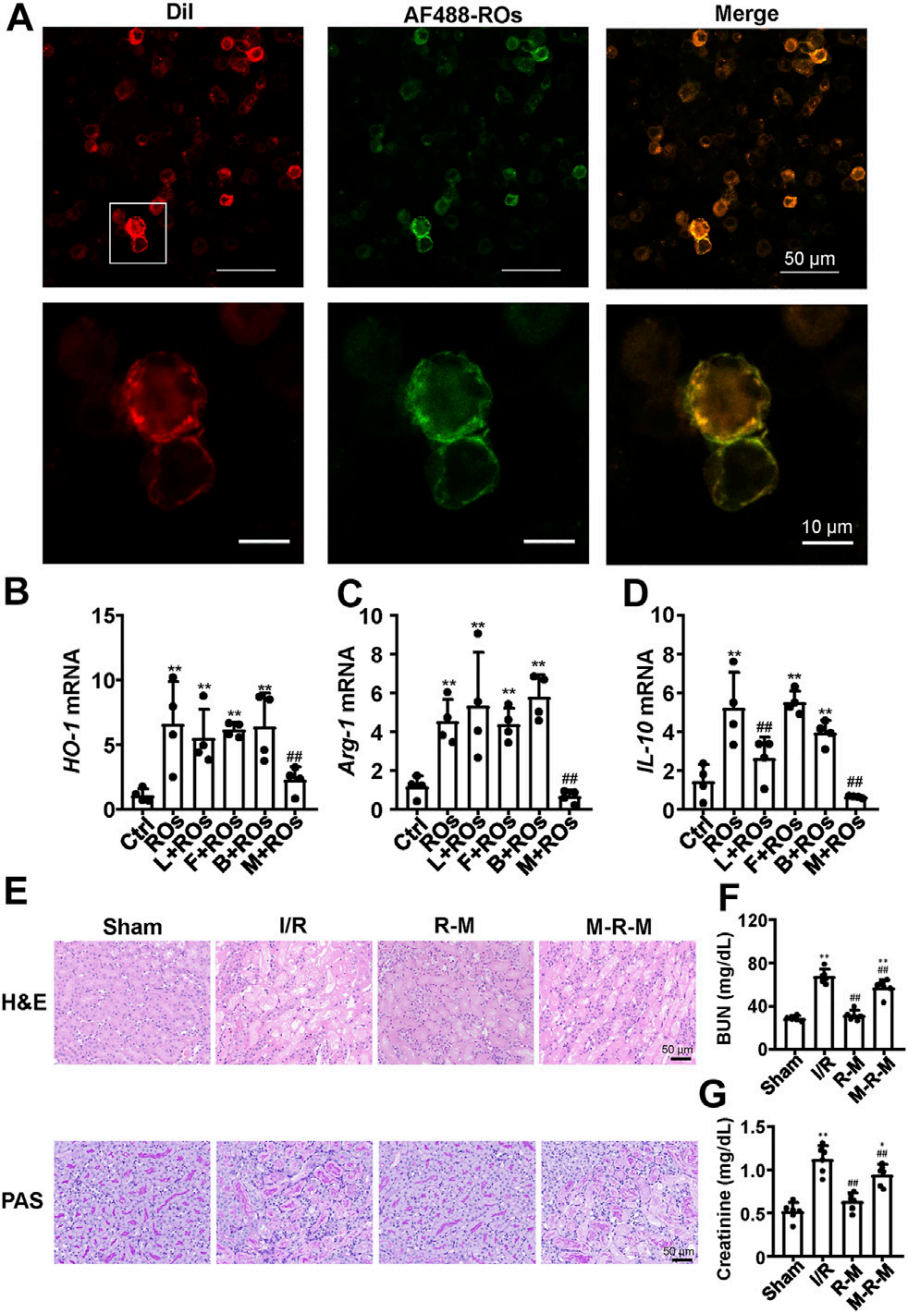
Interfering with mDC-SIGN blocked the riclinoctaose function **(A)** Colocalization of AF488-labeled riclinoctaose and cell membrane dye. Raw 267.4 cells were incubated with AF488-riclinoctaose for 6 h, then strained for confocal microscopy with DiI (red cell membrane dye). **(B–D)** mRNA expression levels of *HO-1*, *Arg-1*, and *IL-10* in BMDMs pretreated with vehicle, laminarin (200 μg/ml), Fcblock (10 μg/ml), bartonella LPS (160 ng/ml), or mannan (200 μg/ml) before riclinoctaose-treatment (300 μM). **(E)** Upper panel: representative kidney section stained for hematoxylin and eosin (H and E) in sham mice, I/R mice, mice treated with riclinoctaose (R–M), and mice treated with riclinoctaose and mannan (M-R-M). Lower panel: representative kidney section stained with Periodic acid–Schiff (PAS) in each mice group. **(F-G)** Blood urea nitrogen (BUN) levels and serum levels of creatinine in each mice group. Ctrl: Control, ROs: Riclinoctaose. Data are expressed as mean ± SD. n = 6, *p < 0.05, **p < 0.01 compared with the sham group or control group; #*p* < 0.05, ##*p* < 0.01 compared with the I/R group or ROs group. Data were analyzed by the two-way ANOVA followed by Bonferroni post hoc analysis.

Then we investigated how riclinoctaose functions on macrophages. Riclinoctaose was conjugated to the AF-488 *via* carbodiimide reagents EDC and NHS. Cultural macrophage cell line Raw 267.4 exhibited strong fluorescence and remained unchanged after further incubation in fresh media for 1 h (Supplementary Figure 3A). On the contrary, AML-12 and NIH-3T3 cells exhibited none of the fluorescence signal (Supplementary Figure 3B,C). To further visualize the localization of riclinoctaose in live cells, AF488-riclinoctaose-treated cells were incubated with a specific dye for the cell membrane. As shown in Figure 7A, the fluorescence of the dye was found to co-localize well with the green channel of AF488-riclinoctaose, suggesting that riclinoctaose bound specifically to the membrane of macrophages. Then, we focused on verifying the interaction of riclinoctaose with macrophages surface receptors. BMDMs were incubated with riclinoctaose for 6 h in the presence of mannans, laminarin, Fcblock, or bartonella LPS, which blocks mDC-SIGN, Dectin-1, FcγR, and TLR4, respectively. Pretreatment of mannan inhibited riclinoctaose-induced HO-1 production by greater than 50% and nearly fully reversed Arg-1 and IL-10 production, and laminarin, Fcblock, or bartonella LPS did not significantly alter the expression of the landmark genes regulated by riclinoctaose (Figures 7B–D). The intravenous injection of BMDMs with co-treatment of riclinoctaose and mannan markedly weaken the protective effect of riclinoctaose on IRI (Figures 7E–G). The above results suggest that mDC-SIGN is potentially responsible for the recognition of the riclinoctaose by mouse macrophages.

## Discussion

Previous evidence has shown that some oligosaccharides exert various biological effects, including anti-inflammatory, antioxidant capacity, and antibacterial (Sutthasupha and Lungkaphin, 2020; Wang et al., 2020; Bi et al., 2021; He et al., 2021). However, these studies were limited to that the vast majority of oligosaccharides are mixture contained oligosaccharides with different degrees of polymerization. Riclinoctaose is an octasaccharide with a definite chemical structure (Wang et al., 2021). Different from other polysaccharides or oligosaccharides, riclinoctaose has great potential as a drug molecular because the production quality of riclinoctaose can be controlled precisely. In this study, we demonstrated the protective effect of riclinoctaose against severe IRI. The protective effects of riclinoctaose were evidenced by multiple criteria, including decreased tubular injury and decreased plasma BUN, creatinine, and lactate dehydrogenase, as well as inhibition of markers of inflammation, fibrosis, and apoptosis. In addition, riclinoctaose promotes M2 polarization of kidney-infiltrating macrophages. Intravenous injection with riclinoctaose-treated macrophages could essentially mimic the therapeutic effects of riclinoctaose *in vivo*. The inhibition of the Nrf-2/HO-1 pathway blocked the renoprotective effects of riclinoctaose. These findings provided substantial evidence that the therapeutic effect of riclinoctaose is primarily *via* the polarization of macrophages.

Macrophage is a heterogeneous cell population that undergoes M1 or M2 activation in response to extracellular cues (Sica and Mantovani, 2012). A biphasic effect of mononuclear phagocytes after IRI has been reported (Lee et al., 2011). Early infiltrating M1 macrophages are mainly responsible for the clearance of apoptotic cells and sublethally injured cells, which may worsen the initial level of kidney injury and further impair glomerular filtration (Lee et al., 2011; Clements et al., 2016). There is a subsequent switch to an alternatively activated M2 macrophage phenotype, which modulates the immune response to promote repair and fibrosis (Murray and Wynn, 2011; Sica and Mantovani, 2012). Riclinoctaose mainly acts during the pro-inflammatory phase of the renal I/R model and promotes earlier myeloid-derived macrophage transformation to an anti-inflammation phenotype, which finally leads to a reduction in renal injury.

Nrf-2 is the upstream regulator of cellular responses against environmental stresses, and itself has been demonstrated to mediate inhibition of the inflammatory cytokine gene expression in M1 macrophages (Kobayashi et al., 2016). Besides, Nrf-2 induces the expression of detoxification and antioxidant enzymes, such as HO-1. There are multiple reports that increased HO-1 exhibits critical immunomodulatory functions in macrophages (Ferenbach et al., 2010; Bolisetty et al., 2017; Zhang et al., 2018). HO-1 catalyzes the degradation of pro-oxidant heme into iron, carbon monoxide, and biliverdin. The anti-inflammatory and cytoprotective effects of HO-1 have been previously observed in different models of renal disease, often hindered by the lack of an appropriate HO-1 inducer (Bolisetty et al., 2017; Zhang et al., 2018). Our results revealed that riclinoctaose is an excellent inducer of HO-1 and promotes the polarization of M2 macrophages. Riclinoctaose-treated macrophages also increased IL-10 mRNA production, a potent anti-inflammatory cytokine reported to inhibits the production of iNOS in macrophages and protects against renal ischemia-induced injury (Deng et al., 2001). The beneficial effects of IL-10 expression are mediated by HO-1 (Lee and Chau, 2002; Chen et al., 2005).

Riclinoctaose binds specifically to the membrane of macrophages but not parenchymal cells or fibroblasts, and riclinoctaose requires mDC-SIGN to induce Nrf-2/HO1-dependent M2 polarization. The interfering of riclinoctaose/mDC-SIGN blocked the function of riclinoctaose on M2 polarization, consequently impairing the renoprotective effect of riclinoctaose. hDC-SIGN was reported to be highly expressed on the population of myeloid cells (Geijtenbeek et al., 2000), and it has turned out to be a target for developing antiviral drugs since it can interact with viral glycans to facilitate virus spreading and exacerbates inflammatory reactions (Jarvis et al., 2019; Fittolani et al., 2021; Ramos-Soriano and Rojo, 2021). hDC-SIGN has been found to be involved in pattern recognition of a broad range of pathogen-derived ligands, meditation of intercellular adhesion, and self-glycoproteins receptor (van Kooyk and Geijtenbeek, 2003; Garcia-Vallejo and van Kooyk, 2013; Jarvis et al., 2019). Recently, hDC-SIGN has gained considerable attention for its participation in the infection process of several pathogens (Fittolani et al., 2021; Ramos-Soriano and Rojo, 2021). Mouse DC-SIGN is one of a family of C-type lectin genes syntenic and homologous to human DC-SIGN. Though there are significant differences in the fine specificity of the C-type lectin domains between mDC-SIGN and hDC-SIGN, mDC-SIGN shares the majority of functions of hDC-SIGN (Garcia-Vallejo and van Kooyk, 2013). There are no previous reports on the hDC-SIGN/mDC-SIGN function in IRI, neither ongoing clinical or preclinical studies based on the therapeutic potential of hDC-SIGN. Riclinoctaose is a promising candidate for clinical studies for protecting renal IRI.

Though we have revealed the potential riclinoctaose/mDC-SIGN as the therapeutic strategy of IRI, future studies to identify a series of oligosaccharides targeting DC-SIGN will be critical. Beyond the Nrf-2/HO-1 pathway that has been thoroughly studied in this study, further exploration will be required to understand detailed mechanistic links mDC-SIGN and IRI. These studies may help the broader therapeutic potential of mDC-SIGN as a target in IRI treatment and present comprehensive therapeutic effects. In summary, riclinoctaose exhibited excellent anti-inflammatory properties *in vitro* and *in vivo*, which may provide further insights into riclinoctaose as an attractive therapeutic agent for inflammatory-related diseases. The identified effect of riclinoctaose/mDC-SIGN could open a new therapeutic avenue for renal IRI by modulated the polarization of M2 macrophages and guide the future development of effective therapies by exploring the immunomodulatory activity of carbohydrates targeting mDC-SIGN.

## Conclusion

**FIGURE 8:**
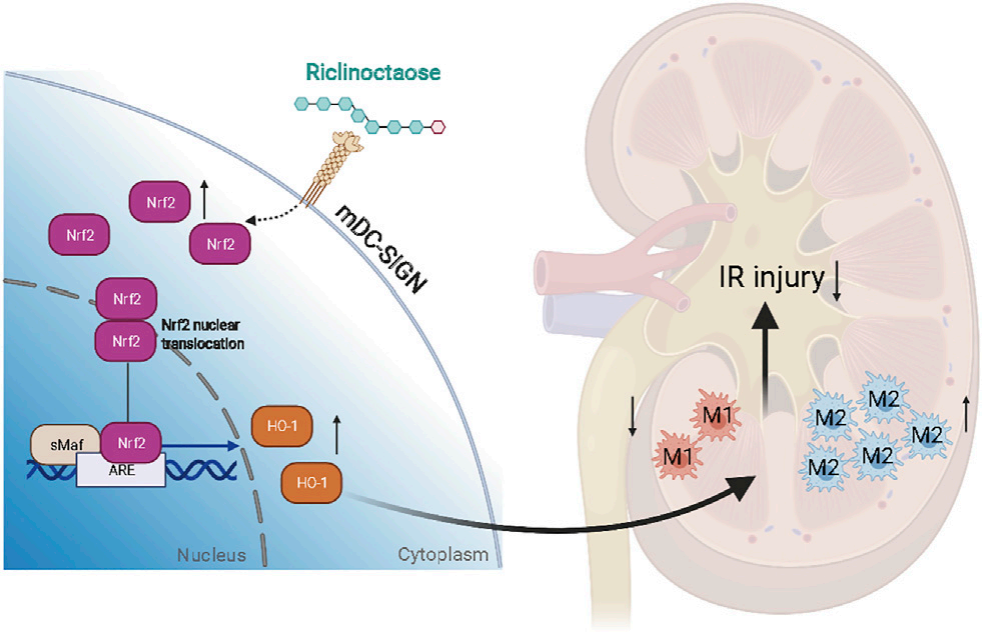
A schematic summary of this study, showing that riclinoctaose protects against renal ischemia-reperfusion injury in mice. In detail, riclinoctaose activates the Nrf-2/HO-1 pathway, inhibits ischemia-reperfusion-induced M1 macrophage polarization, and facilitates M2 macrophage recruitment into the kidneys.

Our results demonstrated that riclinoctaose might be a potential candidate agent for the treatment of renal IRI (Figure 8). The polarization of macrophages induced by Nrf-2/HO-1 activation was demonstrated to regulate the renoprotective effect of riclinoctaose and mDC-SIGN is potentially responsible for the recognition of the riclinoctaose by mouse macrophages.

## Data Availability

The original contributions presented in the study are included in the article/Supplementary Material, further inquiries can be directed to the corresponding authors.
