# Supplementary material for: Riclinoctaose Attenuates Renal Ischemia-Reperfusion Injury by the Regulation of Macrophage Polarization
Source: Front Pharmacol. 2021 Oct 13;12:745425. doi: 10.3389/fphar.2021.745425 (PMC8548467; doi:10.3389/fphar.2021.745425)
Supplement: Supplementary file 1 [file DataSheet1.pdf]

Supplementary Material

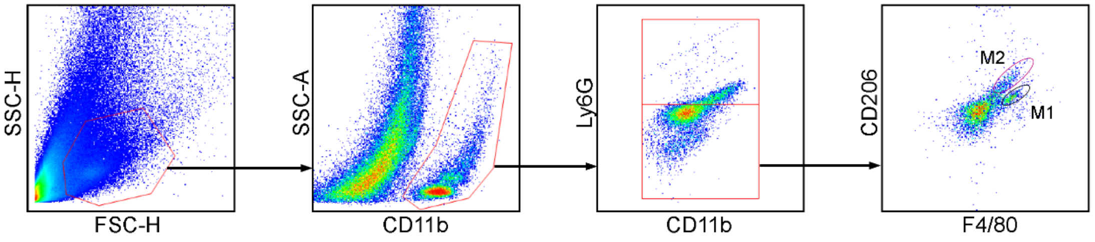

Supplementary Figure 1. Representative flow cytometry gating and analysis strategy.

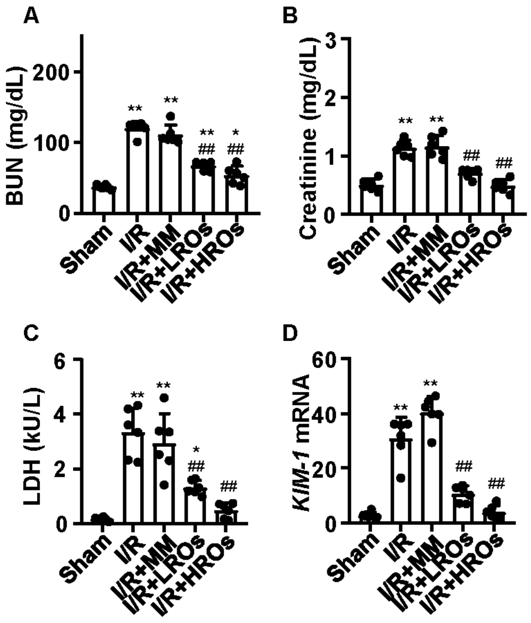

Supplementary Figure 2. Functional protection from renal IRI with riclinoctaose at 48 h after reperfusion. (A-C) Blood urea nitrogen (BUN) levels (A), serum levels of creatinine (B), and lactate dehydrogenase (LDH) (C) in each mice group. **(D)** *Kim1* mRNA levels in kidneys of the four groups of mice. ROs: Riclinoctaose. Data are expressed as mean ± SD. n = 6, *p < 0.05, **p < 0.01 compared with the sham group; #p < 0.05, ##p < 0.01 compared with the I/R group. Data were analyzed by the two-way ANOVA followed by Bonferroni post hoc analysis.

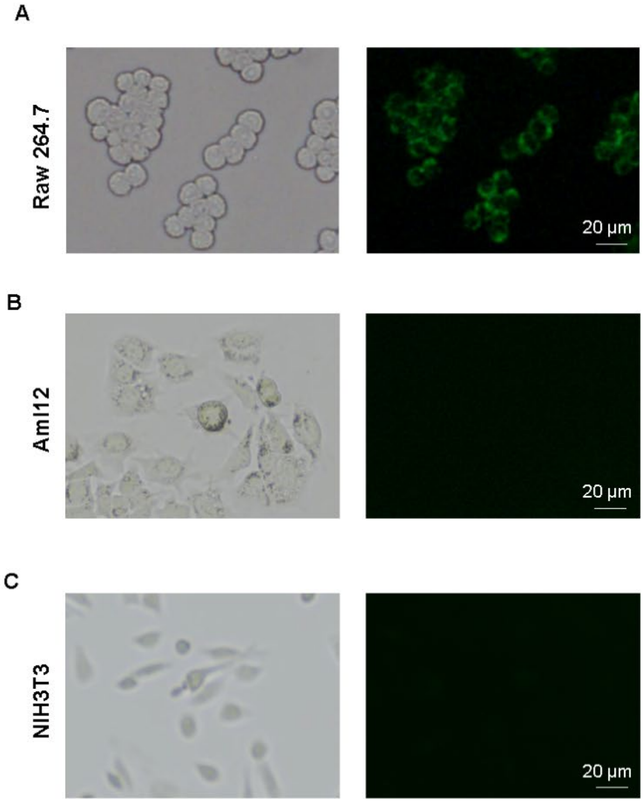

Supplementary Figure 3. Phase-contrast micrographs and fluorescence photomicrographs of Raw264.7 **(A)**, AML12 **(B)**, and NIH3T3 **(C)**.

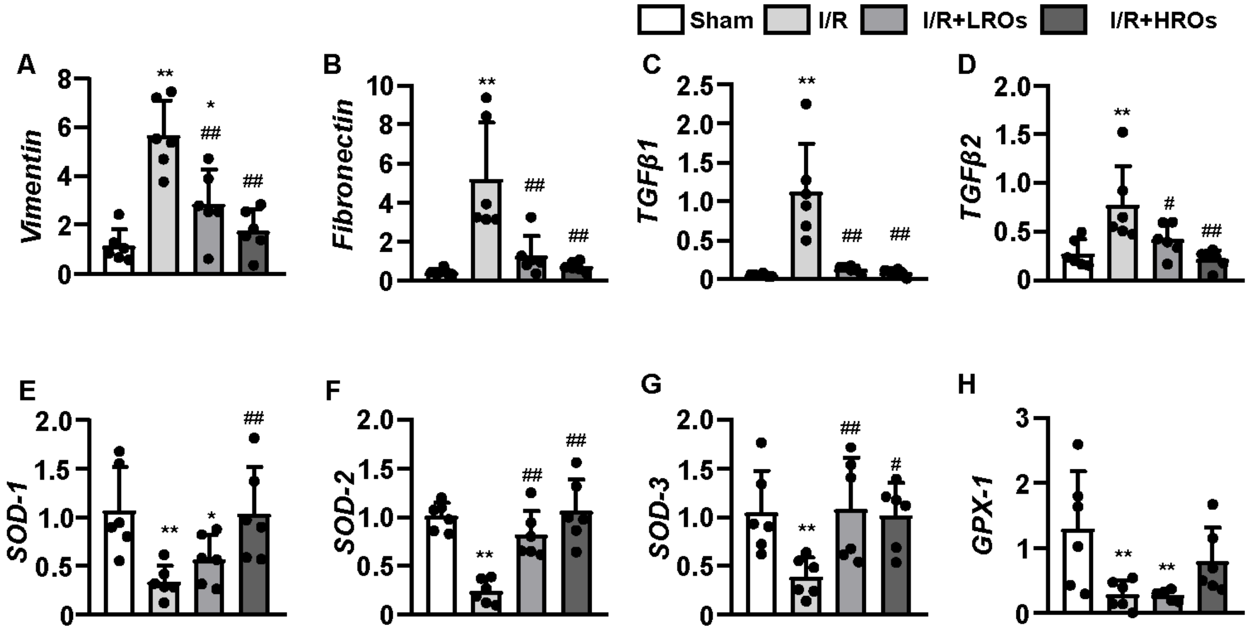

Supplementary Figure 4. Suppression of I/R-induced fibrosis and oxidative stress with riclinoctaose. (A-D) mRNA levels of fibrotic genes, *Vimentin* (A), *Fibronectin* (B), *TGF-β1* (C), and *TGF-β2* (D) in kidneys of the sham group, I/R group, I/R+LROs group, and I/R+HROs group. **(E-H)** mRNA levels of cellular antioxidant enzymes, *SOD-1* (E), *SOD-2* (F), *SOD-3* (G), and *GPX-1* (H) in kidneys of each mice group. The expression of the target gene was normalized to mouse housekeeping gene *Hprt*.
